# Resting-State fMRI in Studies of Acupuncture

**DOI:** 10.1155/2021/6616060

**Published:** 2021-03-23

**Authors:** Xiaoling Li, Lina Cai, Xiaoxu Jiang, Xiaohui Liu, Jingxian Wang, Tiansong Yang, Feng Wang

**Affiliations:** ^1^First Affiliated Hospital, Heilongjiang University of Chinese Medicine, Harbin, China; ^2^Heilongjiang University of Chinese Medicine, Harbin, China

## Abstract

Research exploring the mechanism of acupuncture has been a hot topic in medicine. Resting-state functional magnetic resonance imaging (rs-fMRI) research is a noninvasive and extensive method, which is aimed at the research of the mechanism of acupuncture. Researchers use fMRI technologies to inspect the acupuncture process. The authors reviewed the application of rs-fMRI in acupuncture research in recent 10 years from the aspects of studying acupoints, subjects, acupuncture methods, and intensities. The results found that the application of rs-fMRI in acupuncture research mainly includes research on the onset mechanism of acupuncture treatment; visual evidence of diagnosis and treatment of dominant diseases; efficacy assessments; physiological mechanism of acupoint stimulation; and specific visualization of acupoints.

## 1. Introduction

Acupuncture has been used as a traditional treatment method and is now becoming popular rapidly in clinical medicine practice because of its undeniable therapeutic effects [1, 2]. However, the effect of the acupuncture mechanism on the central system is still unclear, so the exploration of the acupuncture efficacy mechanism has been a hot topic in the medical study [3, 4]. Nowadays, resting-state fMRI is a prominent technique to measure the activities in the brain caused by acupuncture [5, 6].

The objective of this study is to develop an artificial intelligence analysis algorithm and automatic result output system with the function of reading and analyzing fMRI images in acupuncture research. Based on this demand, we preliminarily collected the contents mentioned in this review.

## 2. Comparison of Acupoints

### 2.1. Comparison of a Single Acupuncture Acupoint vs. Acupoint or Nonacupoint

Eight recent studies showed that acupuncture at different acupoints could induce different activities of brain regions in resting-state networks (Table 1). Zhong et al. [7] found that the connectivity between the superior temporal gyrus and anterior insula was enhanced by acupuncture at GB40 and the connection between the STG and postcentral gyrus was increased following acupuncture at KI3. Qiu et al. [8] reported the differences in wavelet transform coherence characteristic curves in the declive, precuneus, postcentral gyrus, supramarginal gyrus, and occipital lobe with acupuncture at LR3 and nonacupoint, and the posteffect can last for 5 minutes. Feng et al. [9] found that compared to nonacupoint, the increase in correlations for acupoints was related with the limbic/paralimbic and subcortical regions and the decrease in correlations was related with the sensory and frontal cortex. Long et al. [10] found that the areas with significant changes in functional connection values after acupuncture at ST36 were mainly concentrated in the middle temporal gyrus and parahippocampal gyrus, which are the main hubs of the default network. The therapeutic effect of acupuncture on pain may be related to the enhanced connection between these two areas. Yu et al. [11] observed that acupuncture at LR3 mainly activated the brain functional network in visual function, associative function, and emotional cognition, which was consistent with the therapeutic effect of LR3 in traditional medicine; furthermore, it had specific values to interpret the acupoint specificity of the LR3. Cai et al. [12] found that needling at the back-shu and front-mu points of the stomach can induce the ReHo changes in the thalamus, the posterior cingulate gyrus, and the precuneus brain regions compared with the single point, and these areas are the important brain regions for points to regulate gastric motility. Nierhaus et al. [13] considered that the stimulation about ST36 can obviously modulate somatosensory and saliency processing activities than nonacupoint. Liu et al. [14] addressed that the combined acupoints activate a wider range of brain areas than single points. In addition, the association of acupoints could activate a few new brain areas and engender new curative effects.

### 2.2. Comparison of before Acupuncture vs. after Acupuncture

More and more results suggested the brain activity after acupuncture was different from that before acupuncture, needing at points could change inherent activities of the cerebral cortex (Table 2). Zhang et al. [15] revealed that needling at LR3 and KI3 specifically promoted the function of brain areas, which are related to vision, emotion, and cognition, and inhibited associated with attention, phonological, and semantic processing. Zhou et al. [16] found that acupuncture at the points of the Lung Meridian could significantly strengthen ALFF in the right gyrus subcallosum and inferior frontal gyrus and weaken in the right postcentral gyrus, left precuneus, superior temporal gyrus, and middle temporal gyrus and needling at the Lung Meridian points could alter inherent activities of the cerebral cortex. Liu et al. [17] observed that compared with before acupuncture, the ReHo in cognitive network, motor network, default network, and limbic system encephalic changed after acupuncture at GB34.

### 2.3. Comparison of Verum Acupuncture vs. Sham Acupuncture

Table 3 presents that verum acupuncture was superior to sham acupuncture. Verum acupuncture stimulated acupoints to produce the sensation of Deqi. Zyloney et al. [18] reported that verum acupuncture (ACU) generated the connectivity in the PAG, PCC, and precuneus, contrasting with sham ACU, and significantly promoted the changes of functional connectivity regions in the pain matrix and default mode network by acupuncture at LR3. Zhao et al. [19] indicated that a true acupuncture effect had more extensive and more significant brain reactions in the long-term stimulation than sham ACU. Adopting the ALFF and ReHo approach, Wu et al. [20] observed that acupuncture at LR3 can modulate the activities of functional brain regions, such as vision, movement, sensation, emotion, and analgesia, but sham ACU does not show correlations in regions with functions. Liu et al. [21] found that poststimulus effects showed a more significant characteristic through verum ACU, and the insula plays a crucial role in the switch process of immediate- and delayed-effect neural responses of acupuncture.

### 2.4. Comparison of Contra-Acupuncture vs. Ipsiacupuncture

The authors used contra-acupuncture vs. ipsiacupuncture to measure the mechanism of acupoints (Table 4). Using ReHo as the observation index, Zhang et al. [22] found that ACC plays an essential role in the regulation of contralateral ST38, on the other pathway of the brainstem-thalamus-cortex, which is an important region on the ipsilateral ST38. Yan et al. [23] revealed the different changes of brain functional connectivity modes after acupuncture at contralateral or ipsilateral ST38, which supported the hypothesis of acupoint specificity.

Two studies designed the comparison of individual differences. Yang et al. [24] found the decreased connectivity in the left middle frontal gyrus in Val/Val homozygous subjects compared with the Val/Val homozygous subjects. The change of ReHo in different brain regions may be related to different constitution groups, and the acupuncture feeling of physical differences may be an important factor affecting acupuncture analgesia [25].

## 3. Comparison of Different Acupuncture Methods

Ten studies to investigate the verity of brain activities used different acupuncture methods (Table 5). The DMN was observed to have a more extensive connectivity following MA and EA, and the connectivity in the sensorimotor network was specifically increased by TEAS [26]. Lv et al. [27] found the changes in the ReHo or ALFF value in brain region functions related to cognitive after-laser acupuncture at TGA. Jiang et al. [28] reported more prominent connectivity between the DMN and the SMN during 30 minutes transcutaneous electrical nerve stimulation (TEAS) compared with MTEAS. Motor function regions connectivity was changed by the xingnaokaiqiao method [29]. Chung et al. [30, 31] pointed out that scalp acupuncture can remarkably enhance the regulation system of the brain network involved in cognition and implementation and the correlation with adjacent brain regions. EA of the auricular concha has an instant effect in modulating the brain default mode network in PI patients, and it may be a brain mechanism underlying improvement of PI [32]. An abdominal acupuncture method could improve the functional connectivities in the cognition network [33]. After electrical stimulation of acupoints, the activities in the frontal lobe, cingulate gyrus, and cerebellum had local changes. In addition, the intensity of changes after 15 minutes were higher than 5 minutes, indicating that the effect of EA on brain functional areas was continuous and strong [34]. Compared with SNA, TENS, TNA, or SNA plus SNA + MS methods showed that the most extensive DMN modulation induced by TNA acupuncture methods can be used as a way of regulating brain activity [35].

## 4. Comparison of Different Stimulation Intensities

Two studies compared the impact of needling at different intensities on brain functional connectivity (Table 6). Shi et al. [36] reported that deep acupuncture with Deqi sensation could regulate neural activity at multiple levels, but no one had Deqi sensation when undergoing shallow acupuncture. Increased connectivity in the MPFC/rACC and dorsolateral prefrontal cortex after enhancement acupuncture compared to standard acupuncture in KOA patients certificated the underlying treatment of the KOA brain mechanism was significantly associated with stimulation intensities [37].

## 5. Dominant Diseases

### 5.1. Nervous System Diseases

Table 7 shows research on the onset acupuncture mechanism of diagnosis and treatment of dominant diseases. Zheng et al. [38] found that needling LIV3 and LI4 can regulate the functional activity of cognition-related regions in AD patients. Acupuncture at LIV3 and LI4 can increase the hippocampal connectivity in AD disease [39]. Wang et al. [40] demonstrated that acupuncture can achieve antidepressant treatment through modulating limbic system activity, particularly the amygdala and the ACC. The changes of DMN connectivity can be used to monitor CM and acupuncture modulate effects after acupuncture [41]. Stroke patients showed decreased FC in the motor cortex, and the decreased FC was increased after acupuncture [42]. Acupuncture at motor-implicated acupoints specifically modulates the motor-related network in stroke patients and enhances the connectivity between the cerebellum and primary sensorimotor cortex. What's more, acupuncture at motor-implicated acupoints could compensate for the decreased connectivity between the cortex and subcortical areas, thus improving motor coordination and subcortical motor learning ability in stroke patients [43]. Tan et al. [44] indicated that acupuncture at tiaoshenyizhi acupoints can improve cognitive function in mild cognitive impairment disease. Chen et al. [45] demonstrated that ACU at TE5 increased the connectivity of the bilateral sensorimotor networks in ischemic stroke patients. Wang et al. [40] included the action of acupuncture on antidepressant patients may be actualized through regulating the areas of the limbic system. After acupuncture at GB34, the functional connectivity between the PM/SMA and SMG was enhanced in stroke patients, suggesting that acupuncture delays the progression of the disease by increasing the communication connection between the damaged white-matter tracts. [46]. Li et al. [47] observed decreased FC in the RFPN could be reversed by acupuncture. Compared with healthy controls, the connectivity between ACC and PCC was enhanced after acupuncture, and the functions of ACC and PCC were associated with cognitive and motor ability that could be interpreted the modulatory effects of acupuncture [48].

### 5.2. Motor System Diseases

Imaging studies evidenced that acupuncture may achieve its therapeutic effect through regulating the connectivity of DMN, salience, central executive, and sensorimotor networks in chronic pain patients [49–51]. Wu et al. [52] showed that acupuncture causes extensive inactivation of almost all limbic systems, pain systems, and DMN in patients with acute lower back pain, which indicated that multiplex networks involved in the treatment of motor system diseases by acupuncture.

### 5.3. Other Diseases

Bao et al. [53] found that the efficacy of acupuncture on CD may involve the regulation of the afferent processing network and DMN in the brain. The effects of acupuncture in treating smoking craving were remarkable, and the SN played an important role in the treatment course [54]. The efficacy of LR3 in connectivity was more concentrated in the frontal lobe, cerebellum, and insula [55]. Pang et al. [56] found that the aberrant neural activity of PMS patients could be regulated by acupuncture at SP6. Acupuncture may adjust the cardiovascular system through multiple brain networks with the cortical level, the hypothalamus, and the brainstem [57].

## 6. Conclusions

This review demonstrates the application of rs-fMRI in the study of the acupuncture mechanism from the aspects of study acupoints, subjects, acupuncture methods, and intensities and found that the application of rs-fMRI in acupuncture research mainly includes research on the onset mechanism of acupuncture treatment; visual evidence of diagnosis and treatment of dominant diseases; efficacy assessments; physiological mechanism of acupoints stimulation; and specific visualization of acupoints. Specifically, the following conclusions can be drawn from the physiological mechanism of acupuncture at acupoints and its specificity research: there are differences in the brain connectivity and local activities between single and different acupoints, and combination points have more wide activate areas than single point; the regulate areas are mostly related to the emotional, cognitive, and painful functions; limbic system and subcortical areas are found to be hubs after acupuncture; verum acupuncture may increase DMN, PAG, PCC, and pain matrix connectivity compared with sham acupuncture, and sham acupuncture influenced less functional areas than true acupuncture; the local brain functional activities of ipsilateral acupuncture are different from those of contralateral acupuncture; the effect of acupuncture has an obvious individual difference; and there are different degrees of changes in brain functional connectivity among different acupunctures, intensities, methods, and different subjects, and an adjusting acupuncture approach can be used as a means of regulating brain activity. In addition, in the study of the onset mechanism of acupuncture treatment, diagnosis and treatment of dominant diseases, and curative effect evaluation, we can see that the brain functional connectivity is not the same in several dominant diseases, acupuncture can treat diseases by regulating the brain network related to cognitive and motor functions, rs-fMRI has an important meaning for the evaluation of the therapeutic effect after acupuncture, and there is a great significance for the establishment of disease-specific biomarkers.

A good technical platform has been offered by the development of medical imaging for the study of acupuncture mechanisms. With the help of fMRI, the inherent and spontaneous neural activities of neurons can be observed from the microscale, mesoscale, and the entire brain area, and it will contribute to depict the organization and mechanism of the brain. However, the sample size is relatively small. In the future, we will collect more samples to expand the database for developing an artificial intelligence algorithm and automatic result output system of fMRI images to explore the cerebral mechanism of acupuncture.

## Figures and Tables

**Table 1:** Research studies on a single acupuncture acupoint vs. acupoint or nonacupoint.

Acupoints	Main findings
GB40 vs. KI3 [7]	Superior temporal gyrus (STG), auditory network, and anterior insula vs STG and postcentral gyrus
LR3 vs. nonacupoint [8]	Declive, precuneus, postcentral gyrus, supramarginal gyrus, and occipital lobe; post-ACU can lasted for 5 minutes
ST36 vs. nonacupoint [9]	Limbic/paralimbic regions
ST36 vs. nonacupoint [10]	Parahippocampal gyrus and middle temporal- major hubs of the default mode network
LR3 vs. nonacupoint [11]	Network: visual, emotion, and cognition
Back-shu + front-mu vs. single [12]	ReHo: thalamus, the posterior cingulate gyrus, and the precuneus
ST36 vs. nonacupoint [13]	Somatosensory and saliency processing regions
Combinations vs. single [14]	More widely activate areas; new brain areas; and curative effects

**Table 2:** Research studies on before acupuncture vs. after acupuncture.

Study	Main findings
Zhang et al. [15]	ALFF: increased cerebral occipital lobe and middle occipital gyrus
	Decreased gyrus rectus of the frontal lobe and posterior lobe
Zhou et al. [16]	ALFF: right gyrus subcallosum, postcentral gyrus, inferior frontal gyrus left precuneus, superior temporal gyrus, and middle temporal gyrus
Liu et al. [17]	ReHo: cognitive, motor, default network, and limbic system

**Table 3:** Research studies on verum acupuncture vs. sham acupuncture.

Study	Main findings
Zyloney et al. [18]	PAG, PCC, and precuneus; pain matrix and default mode network
Zhao et al. [19]	More extensive and remarkable cerebral response
Wu et al. [20]	ALFF + ReHo: vision, movement, sensation, emotion, and analgesia
Liu et al. [21]	Insula

**Table 4:** Research studies contra-acupuncture vs. ipsiacupuncture.

Study	Main findings
Zhang et al. [22]	ReHo: ACC, brainstem-thalamus-cortex
Yan et al. [23]	Increased: anterior cingulate and insula
	Decreased: anterior/paracingulate cortex

**Table 5:** Research studies on different acupuncture methods.

Methods	Main findings
MA + EA, TEAS [26]	MA + EA: more secure and spatially extended connectivity; TEAS: sensorimotor network
Laser acupuncture [27]	Cognitive functions
TEAS [28]	DMN and SMN
XNKQ acupuncture [29]	Motor function
Scalp acupuncture [30, 31]	Cognition, implementation network, and adjacent brain regions
EA [32]	DMN
Abdominal acupuncture [33]	Allomeric function
EA [34]	Higher intensity at 15 minutes than 5 minutes
SNA, SNA + MS, and TENS [35]	Different acupuncture methods to induce different DMN modulatory effects

**Table 6:** Research studies on different stimulation intensities.

Intensities	Main findings
Deep **vs.** shallow [36]	LPNN and DMN
Enhancement **vs.** standard [37]	MPFC/rACC and dorsolateral prefrontal cortex

**Table 7:** Research studies on different dominant diseases.

Dominant diseases	Main findings
AD [38]	Cognition regions
AD [39]	Hippocampal
Depression [40]	Limbic system, amygdala, and the ACC
CM [41]	DMN
Stroke [42]	Bilateral motor cortex
Stroke [43]	Motor-related network
MCI [44]	Cognition regions
Ischemic stroke [45]	Sensorimotor network
Depression [40]	Limbic system, amygdala, and ACC
Stroke [46]	PM/SMA and SMG RFPN
Migraine [47]	ACC and PCC
Stroke [48] chronic pain [49–51]	Networks: DMN, salience, central executive, and sensorimotor
ALBP [52]	Limbic, pain, attentional and somatosensory system, and DMN
CD [53]	Afferent processing network and DMN
Smoking craving [54]	SN
Hypertension [55]	Frontal lobe, cerebellum, and insula
PMS [56]	Aberrant neural activity
Cardiovascular [57]	Cortical, hypothalamus and brainstem

## Abbreviations

STG: Superior temporal gyrus
ReHo: Regional homogeneity
ALFF: Low-frequency fluctuations
PAG: Periaqueductal gray
PCC: Posterior cingulate cortex
ACC: Anterior cingulate cortex
MA: Manual acupuncture
EA: Electroacupuncture
TEAS: Transcutaneous electrical acupoint stimulation
TGA: Thirteen ghost acupoints
MTEAS: Minimal TEAS
PI: Primary insomnia
DMN: Default mode network
SNA: Single-needle acupuncture
TENS: Transcutaneous electrical nerve stimulation
TNA: Three-needle acupuncture
LPNN: Limbic-paralimbic-neocortical network
MPFC: Medial prefrontal cortex
rACC: Rostral anterior cingulate cortex
KOA: Knee osteoarthritis
CM: Chronic migraine
SMA: Supplementary motor area
SMG: Supramarginal gyrus
RFPN: Right frontoparietal network
PMS: Premenstrual syndrome
